# *N*-(2,4-Dichloro­phen­yl)-2,4-dimethyl­benzene­sulfonamide

**DOI:** 10.1107/S1600536810045563

**Published:** 2010-11-13

**Authors:** P. G. Nirmala, Sabine Foro, B. Thimme Gowda, Hartmut Fuess

**Affiliations:** a Department of Chemistry, Mangalore University, Mangalagangotri 574 199, Mangalore, India; b Institute of Materials Science, Darmstadt University of Technology, Petersenstrasse 23, D-64287 Darmstadt, Germany

## Abstract

In the title compound, C_14_H_13_Cl_2_NO_2_S, the mol­ecule is bent at the S atom with an C—SO_2_—NH—C torsion angle of −69.9 (2)°. The dihedral angle between the sulfonyl and aniline benzene rings is 44.0 (1)°. The crystal structure features inversion dimers linked by pairs of N—H⋯O hydrogen bonds. An intra­molecular N—H⋯Cl hydrogen bond is also observed.

## Related literature

For the preparation of the compound, see: Savitha & Gowda (2006 ▶). For our study of the effect of substituents on the structures of *N*-(ar­yl)aryl­sulfonamides, see: Gowda *et al.* (2009 ▶); Nirmala *et al.* (2010**a* ▶,b* ▶). For related structures, see: Gelbrich *et al.* (2007 ▶); Perlovich *et al.* (2006 ▶).
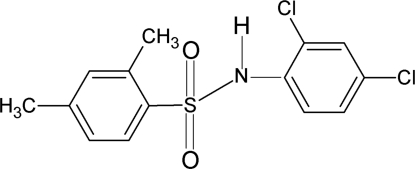


## Experimental

### 

#### Crystal data

C_14_H_13_Cl_2_NO_2_S
*M* *_r_* = 330.21
Triclinic, 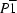

*a* = 8.2407 (9) Å
*b* = 8.3418 (9) Å
*c* = 10.849 (1) Å
α = 84.59 (1)°
β = 89.06 (1)°
γ = 85.07 (1)°
*V* = 739.69 (13) Å^3^
*Z* = 2
Cu *K*α radiation
μ = 5.27 mm^−1^
*T* = 293 K
0.45 × 0.40 × 0.13 mm

#### Data collection

Ennraf–Nonius CAD-4 diffractometer
Absorption correction: ψ scan (North *et al.*, 1968 ▶) *T*_min_ = 0.200, *T*_max_ = 0.547
5173 measured reflections
2629 independent reflections
2496 reflections with *I* > 2σ(*I*)
*R*_int_ = 0.033
3 standard reflections every 120 min  intensity decay: 1.0%

#### Refinement

*R*[*F* ^2^ > 2σ(*F* ^2^)] = 0.056
*wR*(*F* ^2^) = 0.159
*S* = 1.05
2629 reflections
187 parameters
1 restraint
H atoms treated by a mixture of independent and constrained refinement
Δρ_max_ = 0.41 e Å^−3^
Δρ_min_ = −0.80 e Å^−3^

### 

Data collection: *CAD-4-PC* (Enraf–Nonius, 1996 ▶); cell refinement: *CAD-4-PC*; data reduction: *REDU4* (Stoe & Cie, 1987 ▶); program(s) used to solve structure: *SHELXS97* (Sheldrick, 2008 ▶); program(s) used to refine structure: *SHELXL97* (Sheldrick, 2008 ▶); molecular graphics: *PLATON* (Spek, 2009 ▶); software used to prepare material for publication: *SHELXL97*.

## Supplementary Material

Crystal structure: contains datablocks I, global. DOI: 10.1107/S1600536810045563/bq2251sup1.cif

Structure factors: contains datablocks I. DOI: 10.1107/S1600536810045563/bq2251Isup2.hkl

Additional supplementary materials:  crystallographic information; 3D view; checkCIF report

## Figures and Tables

**Table 1:** Hydrogen-bond geometry (Å, °)

*D*—H⋯*A*	*D*—H	H⋯*A*	*D*⋯*A*	*D*—H⋯*A*
N1—H1*N*⋯O2^i^	0.85 (2)	2.24 (2)	3.056 (3)	159 (3)
N1—H1*N*⋯Cl1	0.85 (2)	2.68 (3)	3.020 (2)	105 (2)

Symmetry code: (i) 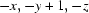.

**Fig. 1.**
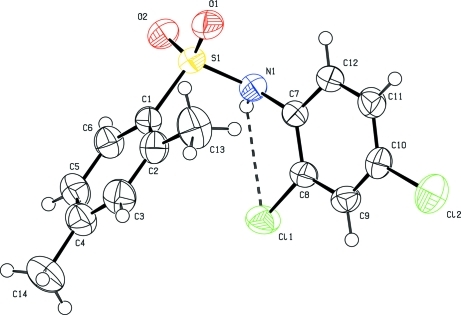
Molecular structure of (I), showing the atom labeling scheme and displacement ellipsoids are drawn at the 50% probability level.

**Fig. 2.**
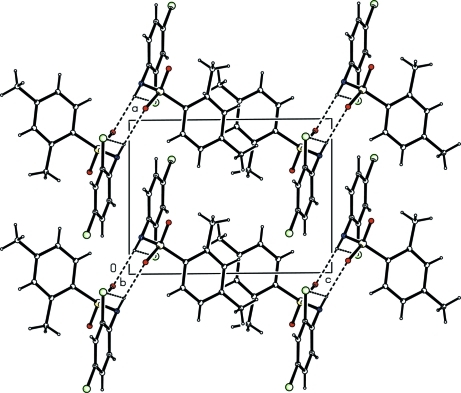
Molecular packing of (I) with hydrogen bonding shown as dashed lines.

## supplementary crystallographic information

### Comment

In the present work, as part of a study of the substituent effects on the structures of *N*-(aryl)arylsulfonamides (Gowda *et al.*, 2009; Nirmala *et al.*, 2010**a*,b*), the structure of 2,4-dimethyl-*N*-(2,4-dichlorophenyl)benzenesulfonamide (I) has been determined (Fig. 1). The molecule is bent at the *N* atom with the C1—SO_2_—NH—C7 torsion angle of -69.9 (2)°, compared to the values of 46.1 (3)° (glide image of molecule 1) and 47.7 (3)° (molecule 2) in the two independent molecules of 2,4-dimethyl-*N*-(phenyl)benzenesulfonamide (II) (Gowda *et al.*, 2009), -54.9 (3)° in 2,4-dimethyl-*N*-(3,5-dichlorophenyl)benzenesulfonamide (III) (Nirmala *et al.*, 2010*b*) and 66.5 (2)° in 2,4-dimethyl-*N*-(2,4-dimethylphenyl)-benzenesulfonamide (IV) (Nirmala *et al.*, 2010*a*)

The two benzene rings in (I) are tilted relative to each other by 44.0 (1)°, compared to the values of 67.5 (1)° (molecule 1) and 72.9 (1)° (molecule 2) in the two independent molecules of(II), 82.3 (1)° in (III) and 41.0 (1)° in (IV). The other bond parameters in (I) are similar to those observed in (II), (III), (IV) and other aryl sulfonamides (Perlovich *et al.*, 2006; Gelbrich *et al.*, 2007).

The structure shows simultaneous N—H···Cl intramolecular and N—H···O intermolecular H-bonding (Table 1). The crystal packing of molecules in (I) *via* N—H···O(S) hydrogen bonds is shown in Fig.2.

### Experimental

The solution of 1,3-xylene (1,3-dimethylbenzene) (10 ml) in chloroform (40 ml) was treated dropwise with chlorosulfonic acid (25 ml) at 0 ° C. After the initial evolution of hydrogen chloride subsided, the reaction mixture was brought to room temperature and poured into crushed ice in a beaker. The chloroform layer was separated, washed with cold water and allowed to evaporate slowly. The residual 2,4-dimethylbenzenesulfonylchloride was treated with 2,4-dichloroaniline in the stoichiometric ratio and boiled for ten minutes. The reaction mixture was then cooled to room temperature and added to ice cold water (100 ml). The resultant solid 2,4-dimethyl-*N*-(2,4-dichlorophenyl)benzenesulfonamide was filtered under suction and washed thoroughly with cold water. It was then recrystallized to constant melting point from dilute ethanol. The purity of the compound was checked and characterized by recording its infrared and NMR spectra (Savitha & Gowda, 2006).

The plate like colorless single crystals used in X-ray diffraction studies were grown in ethanolic solution by a slow evaporation at room temperature.

### Refinement

The H atoms of the NH groups were located in a difference map and later restrained to N—H = 0.86 (2) Å. The other H atoms were positioned with idealized geometry using a riding model with C—H = 0.93–0.96 Å. All H atoms were refined with isotropic displacement parameters (set to 1.2 times of the *U*_eq_ of the parent atom).

### Crystal data

**Table:**

C_14_H_13_Cl_2_NO_2_S	*Z* = 2
*M**_r_* = 330.21	*F*(000) = 340
Triclinic, *P*1	*D*_x_ = 1.483 Mg m^−^^3^
Hall symbol: -P 1	Cu *K*α radiation, λ = 1.54180 Å
*a* = 8.2407 (9) Å	Cell parameters from 25 reflections
*b* = 8.3418 (9) Å	θ = 6.7–18.3°
*c* = 10.849 (1) Å	µ = 5.27 mm^−^^1^
α = 84.59 (1)°	*T* = 293 K
β = 89.06 (1)°	Plate, colorless
γ = 85.07 (1)°	0.45 × 0.40 × 0.13 mm
*V* = 739.69 (13) Å^3^	

### Data collection

**Table:**

Ennraf–Nonius CAD-4 diffractometer	2496 reflections with *I* > 2σ(*I*)
Radiation source: fine-focus sealed tube	*R*_int_ = 0.033
graphite	θ_max_ = 66.9°, θ_min_ = 4.1°
ω/2θ scans	*h* = −9→9
Absorption correction: ψ scan (North *et al.*, 1968)	*k* = −9→9
*T*_min_ = 0.200, *T*_max_ = 0.547	*l* = −12→12
5173 measured reflections	3 standard reflections every 120 min
2629 independent reflections	intensity decay: 1.0%

### Refinement

**Table:**

Refinement on *F*^2^	Secondary atom site location: difference Fourier map
Least-squares matrix: full	Hydrogen site location: inferred from neighbouring sites
*R*[*F*^2^ > 2σ(*F*^2^)] = 0.056	H atoms treated by a mixture of independent and constrained refinement
*wR*(*F*^2^) = 0.159	*w* = 1/[σ^2^(*F*_o_^2^) + (0.1122*P*)^2^ + 0.3056*P*] where *P* = (*F*_o_^2^ + 2*F*_c_^2^)/3
*S* = 1.05	(Δ/σ)_max_ = 0.003
2629 reflections	Δρ_max_ = 0.41 e Å^−^^3^
187 parameters	Δρ_min_ = −0.80 e Å^−^^3^
1 restraint	Extinction correction: *SHELXL97* (Sheldrick, 2008), Fc^*^=kFc[1+0.001xFc^2^λ^3^/sin(2θ)]^-1/4^
Primary atom site location: structure-invariant direct methods	Extinction coefficient: 0.0131 (18)

### Special details

**Table:**

Geometry. All e.s.d.'s (except the e.s.d. in the dihedral angle between two l.s. planes) are estimated using the full covariance matrix. The cell e.s.d.'s are taken into account individually in the estimation of e.s.d.'s in distances, angles and torsion angles; correlations between e.s.d.'s in cell parameters are only used when they are defined by crystal symmetry. An approximate (isotropic) treatment of cell e.s.d.'s is used for estimating e.s.d.'s involving l.s. planes.
Refinement. Refinement of *F*^2^ against ALL reflections. The weighted *R*-factor *wR* and goodness of fit *S* are based on *F*^2^, conventional *R*-factors *R* are based on *F*, with *F* set to zero for negative *F*^2^. The threshold expression of *F*^2^ > σ(*F*^2^) is used only for calculating *R*-factors(gt) *etc*. and is not relevant to the choice of reflections for refinement. *R*-factors based on *F*^2^ are statistically about twice as large as those based on *F*, and *R*- factors based on ALL data will be even larger.

### Fractional atomic coordinates and isotropic or equivalent isotropic displacement parameters (Å^2^)

**Table:**

	*x*	*y*	*z*	*U*_iso_*/*U*_eq_	
C1	0.0771 (3)	0.4275 (3)	0.2785 (2)	0.0417 (5)	
C2	0.1504 (3)	0.3433 (3)	0.3839 (2)	0.0494 (6)	
C3	0.0454 (4)	0.2704 (4)	0.4696 (3)	0.0593 (7)	
H3	0.0903	0.2133	0.5405	0.071*	
C4	−0.1211 (4)	0.2779 (4)	0.4556 (3)	0.0586 (7)	
C5	−0.1883 (4)	0.3659 (4)	0.3526 (3)	0.0571 (7)	
H5	−0.3007	0.3764	0.3428	0.069*	
C6	−0.0905 (3)	0.4382 (3)	0.2642 (2)	0.0497 (6)	
H6	−0.1372	0.4952	0.1939	0.060*	
C7	0.3576 (3)	0.2472 (3)	0.0982 (2)	0.0385 (5)	
C8	0.3155 (3)	0.0907 (3)	0.1315 (2)	0.0394 (5)	
C9	0.4329 (3)	−0.0337 (3)	0.1657 (2)	0.0440 (6)	
H9	0.4039	−0.1381	0.1872	0.053*	
C10	0.5934 (3)	0.0004 (3)	0.1671 (2)	0.0448 (6)	
C11	0.6398 (3)	0.1535 (3)	0.1328 (2)	0.0496 (6)	
H11	0.7490	0.1741	0.1331	0.060*	
C12	0.5214 (3)	0.2753 (3)	0.0980 (2)	0.0459 (6)	
H12	0.5516	0.3785	0.0739	0.055*	
C13	0.3296 (4)	0.3251 (5)	0.4112 (3)	0.0705 (9)	
H13A	0.3685	0.4300	0.4134	0.085*	
H13B	0.3869	0.2698	0.3477	0.085*	
H13C	0.3478	0.2639	0.4899	0.085*	
C14	−0.2268 (5)	0.1923 (5)	0.5518 (4)	0.0833 (11)	
H14A	−0.1703	0.1745	0.6292	0.100*	
H14B	−0.2503	0.0904	0.5250	0.100*	
H14C	−0.3268	0.2579	0.5621	0.100*	
N1	0.2394 (3)	0.3750 (2)	0.06051 (18)	0.0425 (5)	
H1N	0.156 (3)	0.347 (4)	0.026 (3)	0.051*	
O1	0.3348 (2)	0.5697 (2)	0.1953 (2)	0.0564 (5)	
O2	0.0776 (3)	0.6299 (2)	0.08078 (18)	0.0559 (5)	
Cl1	0.11490 (8)	0.04794 (8)	0.12875 (7)	0.0581 (3)	
Cl2	0.74198 (9)	−0.15459 (9)	0.20963 (7)	0.0672 (3)	
S1	0.18758 (7)	0.51721 (7)	0.15244 (5)	0.0428 (3)	

### Atomic displacement parameters (Å^2^)

**Table:**

	*U*^11^	*U*^22^	*U*^33^	*U*^12^	*U*^13^	*U*^23^
C1	0.0409 (12)	0.0415 (13)	0.0434 (12)	−0.0039 (9)	0.0029 (10)	−0.0073 (10)
C2	0.0487 (15)	0.0545 (16)	0.0446 (13)	0.0003 (11)	−0.0017 (11)	−0.0077 (11)
C3	0.0670 (18)	0.0663 (18)	0.0424 (13)	0.0009 (14)	−0.0003 (12)	0.0003 (12)
C4	0.0614 (17)	0.0637 (18)	0.0516 (15)	−0.0101 (14)	0.0112 (13)	−0.0078 (13)
C5	0.0440 (14)	0.0716 (19)	0.0575 (15)	−0.0091 (13)	0.0056 (12)	−0.0119 (13)
C6	0.0450 (14)	0.0559 (15)	0.0475 (13)	−0.0012 (11)	−0.0019 (10)	−0.0034 (11)
C7	0.0397 (12)	0.0382 (12)	0.0374 (11)	−0.0034 (9)	0.0025 (9)	−0.0027 (9)
C8	0.0398 (12)	0.0406 (13)	0.0384 (11)	−0.0086 (9)	0.0006 (9)	−0.0029 (9)
C9	0.0503 (14)	0.0380 (12)	0.0429 (12)	−0.0041 (10)	−0.0009 (10)	0.0008 (9)
C10	0.0440 (13)	0.0455 (13)	0.0439 (12)	0.0053 (10)	−0.0032 (10)	−0.0071 (10)
C11	0.0381 (13)	0.0524 (15)	0.0599 (15)	−0.0058 (11)	0.0026 (11)	−0.0122 (12)
C12	0.0413 (13)	0.0404 (13)	0.0568 (14)	−0.0082 (10)	0.0079 (10)	−0.0058 (10)
C13	0.0512 (17)	0.097 (3)	0.0608 (17)	0.0054 (16)	−0.0101 (13)	−0.0045 (16)
C14	0.084 (3)	0.092 (3)	0.073 (2)	−0.018 (2)	0.0244 (19)	0.0047 (19)
N1	0.0435 (11)	0.0382 (11)	0.0450 (11)	−0.0008 (8)	0.0010 (8)	−0.0015 (8)
O1	0.0508 (11)	0.0462 (11)	0.0749 (12)	−0.0120 (8)	0.0031 (9)	−0.0134 (9)
O2	0.0590 (12)	0.0403 (10)	0.0646 (11)	0.0061 (8)	0.0026 (9)	0.0059 (8)
Cl1	0.0405 (4)	0.0519 (4)	0.0825 (5)	−0.0131 (3)	−0.0042 (3)	−0.0001 (3)
Cl2	0.0586 (5)	0.0630 (5)	0.0758 (5)	0.0180 (3)	−0.0144 (4)	−0.0036 (4)
S1	0.0434 (4)	0.0333 (4)	0.0512 (4)	−0.0023 (2)	0.0033 (3)	−0.0024 (3)

### Geometric parameters (Å, °)

**Table:**

C1—C6	1.387 (4)	C9—C10	1.378 (4)
C1—C2	1.402 (4)	C9—H9	0.9300
C1—S1	1.773 (2)	C10—C11	1.381 (4)
C2—C3	1.393 (4)	C10—Cl2	1.737 (2)
C2—C13	1.502 (4)	C11—C12	1.377 (4)
C3—C4	1.378 (5)	C11—H11	0.9300
C3—H3	0.9300	C12—H12	0.9300
C4—C5	1.374 (4)	C13—H13A	0.9600
C4—C14	1.515 (4)	C13—H13B	0.9600
C5—C6	1.372 (4)	C13—H13C	0.9600
C5—H5	0.9300	C14—H14A	0.9600
C6—H6	0.9300	C14—H14B	0.9600
C7—C12	1.389 (3)	C14—H14C	0.9600
C7—C8	1.393 (3)	N1—S1	1.645 (2)
C7—N1	1.416 (3)	N1—H1N	0.853 (18)
C8—C9	1.384 (3)	O1—S1	1.424 (2)
C8—Cl1	1.722 (2)	O2—S1	1.4311 (19)
			
C6—C1—C2	120.8 (2)	C11—C10—Cl2	119.1 (2)
C6—C1—S1	115.43 (19)	C12—C11—C10	118.7 (2)
C2—C1—S1	123.7 (2)	C12—C11—H11	120.6
C3—C2—C1	115.9 (3)	C10—C11—H11	120.6
C3—C2—C13	118.1 (3)	C11—C12—C7	121.4 (2)
C1—C2—C13	125.9 (3)	C11—C12—H12	119.3
C4—C3—C2	123.9 (3)	C7—C12—H12	119.3
C4—C3—H3	118.1	C2—C13—H13A	109.5
C2—C3—H3	118.1	C2—C13—H13B	109.5
C5—C4—C3	118.2 (3)	H13A—C13—H13B	109.5
C5—C4—C14	121.1 (3)	C2—C13—H13C	109.5
C3—C4—C14	120.7 (3)	H13A—C13—H13C	109.5
C6—C5—C4	120.4 (3)	H13B—C13—H13C	109.5
C6—C5—H5	119.8	C4—C14—H14A	109.5
C4—C5—H5	119.8	C4—C14—H14B	109.5
C5—C6—C1	120.8 (3)	H14A—C14—H14B	109.5
C5—C6—H6	119.6	C4—C14—H14C	109.5
C1—C6—H6	119.6	H14A—C14—H14C	109.5
C12—C7—C8	118.3 (2)	H14B—C14—H14C	109.5
C12—C7—N1	119.8 (2)	C7—N1—S1	120.33 (16)
C8—C7—N1	121.9 (2)	C7—N1—H1N	115 (2)
C9—C8—C7	121.2 (2)	S1—N1—H1N	110 (2)
C9—C8—Cl1	118.61 (18)	O1—S1—O2	119.34 (13)
C7—C8—Cl1	120.21 (19)	O1—S1—N1	106.94 (12)
C10—C9—C8	118.6 (2)	O2—S1—N1	104.56 (11)
C10—C9—H9	120.7	O1—S1—C1	110.04 (12)
C8—C9—H9	120.7	O2—S1—C1	108.11 (12)
C9—C10—C11	121.8 (2)	N1—S1—C1	107.11 (11)
C9—C10—Cl2	119.1 (2)		
			
C6—C1—C2—C3	1.3 (4)	C8—C9—C10—C11	−1.5 (4)
S1—C1—C2—C3	−175.4 (2)	C8—C9—C10—Cl2	−179.91 (18)
C6—C1—C2—C13	−179.0 (3)	C9—C10—C11—C12	1.0 (4)
S1—C1—C2—C13	4.4 (4)	Cl2—C10—C11—C12	179.3 (2)
C1—C2—C3—C4	−0.2 (4)	C10—C11—C12—C7	0.7 (4)
C13—C2—C3—C4	−180.0 (3)	C8—C7—C12—C11	−1.7 (4)
C2—C3—C4—C5	−1.8 (5)	N1—C7—C12—C11	−179.3 (2)
C2—C3—C4—C14	178.7 (3)	C12—C7—N1—S1	−74.9 (3)
C3—C4—C5—C6	2.6 (5)	C8—C7—N1—S1	107.6 (2)
C14—C4—C5—C6	−177.8 (3)	C7—N1—S1—O1	48.1 (2)
C4—C5—C6—C1	−1.6 (4)	C7—N1—S1—O2	175.56 (18)
C2—C1—C6—C5	−0.4 (4)	C7—N1—S1—C1	−69.8 (2)
S1—C1—C6—C5	176.5 (2)	C6—C1—S1—O1	151.8 (2)
C12—C7—C8—C9	1.1 (4)	C2—C1—S1—O1	−31.4 (3)
N1—C7—C8—C9	178.6 (2)	C6—C1—S1—O2	19.9 (2)
C12—C7—C8—Cl1	−178.02 (18)	C2—C1—S1—O2	−163.3 (2)
N1—C7—C8—Cl1	−0.5 (3)	C6—C1—S1—N1	−92.3 (2)
C7—C8—C9—C10	0.5 (4)	C2—C1—S1—N1	84.5 (2)
Cl1—C8—C9—C10	179.62 (18)		

### Hydrogen-bond geometry (Å, °)

**Table:**

*D*—H···*A*	*D*—H	H···*A*	*D*···*A*	*D*—H···*A*
N1—H1N···O2^i^	0.85 (2)	2.24 (2)	3.056 (3)	159 (3)
N1—H1N···Cl1	0.85 (2)	2.68 (3)	3.020 (2)	105 (2)

Symmetry codes: (i) −*x*, −*y*+1, −*z*.
